# Books and Magazines

**Published:** 1905-04

**Authors:** 


### Books and Magazines.

---

Lea's Series of Medical Epitomes.—Edited by Victor C. Pedersen, M. D.—Hollis' Epitome of Medical Diagnosis.—A Manual for Students and Physicians. By Austin W. Hollis, M. D., Attending Physician to St. Luke's Hospital; to the New York Dispensary, etc. In one 12mo volume of 319 pages, with 13 illustrations. Cloth, $1, net. Lea Brothers & Co., Publishers, Philadelphia and New York, 1905.

For the student preparing for examinations, the candidate who is brushing up for appearance before his State Board, or for the physician who wishes a handy volume to slip into his pocket or under the cushion of his carriage seat, so that at odd moments he may refresh his memory on forgotten details or post himself on the most recent knowledge on any medical subject, the volumes of this series have proved to be far in advance of any previous attempt. This is the fifteenth of the series. It naturally does not claim originality, but it embodies an earnest effort to give a clear, accurate, compendious covering of the essentials of its subject, presented with a due sense of the relative importance of its various branches.

---

Essentials of the Practice of Medicine.—Prepared especially for students of medicine. By William R. Williams, M. D., formerly Instructor in Medicine and Lecturer in Hygiene, Cornell University; Tutor in Therapeutics, Columbia University (College of Physicians and Surgeons), New York. 12mo of 461 pages. Philadelphia and London: W. B. Saunders & Company, 1905. Double number. Cloth, $1.75, net.

In this new volume in Saunders' Question-Compend Series the student is provided with a book of the utmost practical value. Throughout the work special stress has been laid on the more common aspects of the various diseases, emphasizing the contrasting points in similar conditions, so as to render differential diagnosis as easy as possible. Symptomatology and treatment have likewise been adequately, although concisely, considered. In fact, this little work is the best we have seen, and for students preparing for examination it will be a most welcome and trusty aid. It contains a vast amount of practical, essential information in the least possible space.

---

The Urine and Feces.—A practical Manual on the Urine and Feces in Diagnosis. By Otto Hensel, Ph. G., M. D., Bacteriologist to the German Hospital, New York, and Richard Weil, A. M., M. D., Pathologist to the German Hospital, New York, in collaboration with Smith Ely Jelliffe, M. D., Ph. D., Instructor in Pharmacology and Therapeutics, Columbia University; Visiting Neurologist, City Hospital, New York. In one octavo volume of 334 pages, illustrated with 116 engravings and 10 colored plates. Cloth, $2.75, net. Lea Brothers & Co., Publishers, New York and Philadelphia, 1905.

Although there are a number of large and exhaustive treatises on clinical laboratory methods of diagnosis, it is believed that this is the first compact, convenient and practical hand-book on the subject. It has been the aim of the authors to supply a trustworthy guide arranged for ready use, and complete enough for the actual daily needs of the working practitioner.

With the rapid growth of the use of precise methods in diagnosis the value of a manual of this kind becomes more and more evident. The authors, from their large hospital experience, are well fitted to furnish the information that is most valuable.

---

STUDIES IN THE PSYCHOLOGY OF SEX—SEXUAL SELECTION IN MAN.—1, Touch; 2, Smell; 3, Hearing; 4, Vision. By Havelock Ellis. 6⅜x8⅞ inches. Pages XII-270. Extra cloth, $2, net. Sold only by subscription to physicians, lawyers, and scientists. F. A. Davis Company, Publishers, 1914-16 Cherry Street, Philadelphia.

This important and long expected work is to be completed in five volumes. Each volume will be sold separately and is complete in itself. It is the only edition in English published by the author's permission.

These books have been so extensively advertised and the author is so famous that expectation has been on tiptoe, and they will be eagerly read. The above is the second of the series. "An Analysis of the Sexual Impulse, Love, and Pain," the first volume, was noticed in our book reviews in January, 1904.

---

SAUNDERS' YEAR-BOOK OF MEDICINE AND SURGERY FOR 1905.—A Yearly Digest of Scientific Progress and Authoritative Opinion in all Branches of Medicine and Surgery, drawn from journals, monographs, and text-books of the leading American and foreign authors and investigators. Arranged, with critical editorial comments, by eminent American specialists, under the editorial charge of George M. Gould, A. M., M. D. In two volumes. Volume I, including General Medicine; Volume II, General Surgery. Two octavos of about 700 pages each, fully illustrated. Philadelphia and London: W. B. Saunders & Co., 1905. Per volume: cloth, $3, net; half Morocco, $3.75, net.

The 1905 issue of Saunders' American Year-Book of Medicine and Surgery fully maintains the pre-eminent position which it long ago established. Dr. Gould, the editor, has associated with him a staff of men of the greatest ability, shown in the conscientious thoroughness with which each article is prepared. Here the practitioner has placed before him, and at a very moderate price, the cream of all the medical literature published during the past year, and in such a form that it is readily digestible. As a compendium of medical and surgical progress, it will prove invaluable; for the practitioner anxious to keep abreast of the advances in the subjects treated, it will be of the utmost assistance. The text, as usual, contains a number of illustrations of practical value; there are also nine insert plates of much excellence.

---

A Text-Book of Clinical Anatomy.—For Students and Practitioners. By Daniel N. Eisendrath, A. B., M. D., Clinical Professor of Anatomy in the Medical Department of the University of Illinois (College of Physicians and Surgeons); Attending Surgeon to the Cook County Hospital, Chicago, etc. Handsome octavo of 515 pages, beautifully illustrated with 153 illustrations, a number in colors. Philadelphia, New York, London: W. B. Saunders & Co., 1905. Cloth, $5, net; sheep or half Morocco, $6, net.

The subject of anatomy, and especially clinical anatomy, is so closely allied to practical medicine and surgery that it is absolutely impossible for a physician or surgeon to practice his profession successfully unless he have an intimate knowledge of the human structure. In his preface the author states that the primary object of his work is to serve as a bridge for both the practitioner and student from descriptive anatomy, as it is usually taught in the first two years of a medical course, to its daily application at the bedside, in the clinic, or in the operating room. The entire subject is discussed with a thoroughness and precision that spring from experience. The method of illustrating the subject is novel, special attention having been given to surface anatomy. The illustrations themselves are the result of a great deal of painstaking study, outlines having been marked upon a normal artist model, and then photographed. They are reproduced in the highest style of art, and show far better than any we have seen the relation of anatomic structures from a clinical standpoint, presenting to the practitioner a picture as met at the bedside, with the skin covering the tissue. The work is indeed magnificent; text, illustrations, paper, typography, and binding being of unusual excellence.

---

Tanner—Memoranda of Poisons and Their Antidotes and Tests. By Thos. Hawkes Tanner, M. D. Ninth edition, revised by Henry Leffmann, M. D., Professor of Chemistry, Woman's Medical College of Pennsylvania; Vice-President Society of Public Analysts. 12mo. Cloth, net, 75 cents.

Eight editions attest the usefulness of this little book, which, without being much increased in size, has always been kept fully up to date, each new edition containing new matters of importance and omitting the obsolete portions of the old text. It will be found a complete manual for ready reference, giving the diagnosis of poisoning, the duties of practitioners in such cases, the treatment of poisoning, determination of poisons and classification of poisons with special descriptions of each drug, its actions and antidotes.

---

A TEXT-BOOK FOR MEDICAL AND LEGAL PRACTITIONERS AND STUDENTS. By John J. Reese, M. D., editor of "Taylor's Jurisprudence," formerly Professor of the Principles and Practice of Medical Jurisprudence, including Toxicology, in the University of Pennsylvania Medical Department. Sixth edition, revised and edited by Henry Leffmann, M. D., Pathological Chemist, Jefferson Medical College Hospital; Chemist, State Board of Health; Professor of Chemistry, Woman's Medical College of Pennsylvania, etc. 12mo. 660 pages. Cloth, net, $3; leather, net, $3.50.

During the publication of its six editions, Reese's Medical Jurisprudence has probably been used more as a student's text-book than any similar work published on this subject. The revision of the present edition has been more thorough than that of any of its predecessors. It is practically a new book; one up to date in every way and as thorough as the student on this subject requires for any regular college course.

"To the student of medical jurisprudence and toxicology it is invaluable, as it is concise, clear and thorough in every respect." —*The American Journal of the Medical Sciences.*

---

THE PRACTICE OF OBSTETRICS. By J. Clifton Edgar, M. D., Professor of Obstetrics and Clinical Midwifery, Medical Department of Cornell University, New York City; Attending Obstetrican to the New York Maternity Hospital, etc. Commended by over 150 prominent instructors and over 50 journals. Second edition, revised, enlarged, improved, with 1264 illustrations (43 more than in first edition), including 5 colored plates (3 more than in first edition), and 38 figures in colors. Royal octavo; 1153 pages (42 more than in first edition). In cloth, $6; in sheep, $7, net.

"The modest title of the 'Practice of Obstetrics' of Dr. Edgar's book might seem to imply that the author does not aspire to instruct his fellow-teachers and specialists, nor expect his book to interest them or to alter their opinions. The student may suppose that it is a mere digest to help him to pass his examination, and the practitioner that it is a handy guide in the common emergencies of practice. It is in reality the most comprehensive treatise on the science and practice of midwifery that has yet appeared. It is distinguished from all previous works on the subject by its extraordinary wealth of illustration. Everything that could possibly be made clearer or plainer by means of illustrations is so illustrated. The book is a great work, the most notable addition to obstetric literature that has appeared in recent years."—*The British Medical Journal.*

---

DISEASES OF THE DIGESTIVE TRACT. By John C. Hemmeter, M. D. Author of "A Treatise on Diseases of the Stomach"; Professor in the Medical Department of the University of Maryland; Consultant to the University Hospital and Director of the Clinical Laboratory. P. Blakiston's Son & Co., Philadelphia. Volume I, 742 pages, 2 plates in colors, copiously illustrated. Volume II, 679 pages, 10 full-page plates and other illustrations. Per volume, cloth, $5; sheep, $6, net.

"Diseases of the Intestines." Two volumes. These books, with Hemmeter on Diseases of the Stomach, form a complete treatise on Diseases of the Digestive Tract. The subject is covered thoroughly and systematically by an author of well-known reputation and ability. The results of recent investigation, by which so much progress has been made in the pathology, diagnosis, and medical and surgical treatment of disorders of the intestinal tract, make their issue at this time of special importance. They are handsomely illustrated, exhaustive, and written for the general practitioner, taking into special consideration American habits of living, diet, and climate.

PRACTICAL MEDICAL SERIES OF YEAR BOOKS. Ten volumes on the year's progress in medicine and surgery. Issued monthly, by the Year Book Publishers, Chicago, under the general editorial charge of Gustavus P. Head, M. D., Chicago P. G. School. SURGERY. (1904). Edited by Jno. B. Murphy, M. D. The price of this volume is $1.50. MEDICINE. (1904). Edited by Drs. Frank Billings and J. H. Salisburn. The price of this volume is $1.

This series is published primarily for the general practitioner, at the same time the arrangement in several volumes enables those interested in special subjects to buy only the parts they desire.

---

THE URINE AND FECES IN DIAGNOSIS. By Otto Hensel, Ph.G., M. D., Bacteriologist, German Hospital, New York, and Richard Weil, A. M., M. D., Bacteriologist German Hospital, New York, in collaboration with Smith Ely Jelliffe, M. D., Ph.D., Columbia University. Illustrated with 116 engravings and 10 colored plates. 334 pages. Cloth, price, not stated. Lea Brothers & Co., New York and Philadelphia.

This book is intended for reference and use in the laboratory and the office when one can not well look up what he wants to know and wants to know it quick, in the larger and exhaustive treatises on the subject. It will be found to be a reliable and trustworthy guide to the study of these excretions so necessary to correct diagnoses.

---

THE DOCTOR'S RECREATION SERIES.—A Book About Doctors. Received January, 1905. Charles Wells Moulton, general editor. By John Gordy Jeaffreson, author of "The Real Lord Byron," "The Real Shelley," etc. 1904. The Saalsfield Publishing Co., Akron, O. Illustrated. Cloth, $2.50.

This is the fourth volume of this delightful series. Beginning with the English doctors of the sixteenth century, the author relates interesting incidents in the life of some of the most noted—Linacre, the first president of the College of Physicians, chartered by old Henry VIII; Mayerne, the great quack, with his Balsam of Bats; about Reynolds, Bulleyn, Butts, and going through the time of all the English kings and queens to George IV, and Victoria, tells about all the noted doctors of the times—Harvey, Hunter, Sydenham, Abernathy, Arbuthnot, et al. It is a storehouse of information during the time when the practice of medicine was purely empirical and humbuggery.

---

History of the Twentieth Tennessee Infantry Regiment, C. S. A. By Dr. W. J. McMurray. Published by the Publication Committee, consisting of Drs. W. J. Murray, Deering J. Roberts, and Ralph Neal, Nashville, Tenn.: 1904.

Part I is devoted to the causes of the war—the right of secession, etc.; the history and personnel of the several companies of the Twentieth, with portraits of those who participated prominently in the scenes of the stirring times. It contains much valuable historical data not elsewhere available. It is a monument to the heroes of the Twentieth—an heirloom for their descendants through all coming ages, which will serve to keep their memories fresh and green. We are indebted to our dear friend, Roberts, an old Confederate surgeon, for this valuable addition to our library.

---

The Wetherill Color Tests—Blood Urine, Feces, Moisture. G. P. Pilling & Son, Philadelphia, Publishers. By Henry Emerson Wetherill, M. D.

This consists of a set of circular color scales by which to compare color of specimens to be examined, and a set of test papers. It is a pocket size. Price, not stated.

---

The Ophthalmic Year-Book.—A Digest of the Literature of Ophthalmology, with Index of Publications for 1903. By Edward Jackson, A. M., M. D., Emeritus Professor Diseases of the Eye, Philadelphia Polyclinic; President American Academy of Ophthalmology, and Oto-Laryngology, etc. 45 illustrations. The Herrick Book and Stationery Co., Denver, Col.: 1904. 260 pages. Price, not stated.

This book is what is indicated in the title,—a digest of the literature of the subject with list of publications for 1903.

